# Facile fabrication of a novel self-healing and flame-retardant hydrogel/MXene coating for wood

**DOI:** 10.1038/s41598-023-28228-5

**Published:** 2023-02-01

**Authors:** Xiaojiong Zhao, Min Tian, Ruichao Wei, Saihua Jiang

**Affiliations:** 1 Institute of Safety Science and Engineering, School of Mechanical and Automotive Engineering, South China University of Technology, Wushan Road 381, Guangzhou, 510641 People’s Republic of China; 2 Guangdong Provincial Key Laboratory of Technique and Equipment for Macromolecular Advanced Manufacturing, South China University of Technology, Guangzhou, 510641 People’s Republic of China; 3 State Key Laboratory of Fire Science, University of Science and Technology of China, Jinzhai Road 96, Hefei, 230026 People’s Republic of China; 4 Research Institute of New Energy Vehicle Technology, Shenzhen Polytechnic, Shenzhen, 518055 Guangdong People’s Republic of China; 5 School of Automobile and Transportation, Shenzhen Polytechnic, Shenzhen, 518055 Guangdong People’s Republic of China

**Keywords:** Soft materials, Nanoparticles, Two-dimensional materials

## Abstract

To improve flame retardancy of wood, a novel high-water-retention and self-healing polyvinyl alcohol/phytic acid/MXene hydrogel coating was developed through facile one-pot heating and freeze–thaw cycle methods, and then painted on wood surface. The coating exhibit excellent self-healing property and significantly enhanced water-retention property (water content ≥ 90 wt%), due to the increased hydrogen bonds within the coating system with the presence of MXene nanosheets. Compared to pristine wood, the flame retardancy of coated wood is greatly improved, such as passed V0 rating in UL-94 test, increasing time to ignition (TTI, from 32 to 69 s), and decreased heat release rate and total heat release by 41.6% and 36.14%. The cooling effect and large thermal capacity of high-water-retention hydrogel, and physical barrier effects for flammable gas products, heat and oxygen by MXene nanosheets and the compact char layer formed during combustion play key roles in the flame retardancy enhancements of the wood. High thermal stability of MXene nanosheets is another beneficial factor. The detailed flame-retardant and self-healing mechanisms were proposed.

## Introduction

As a green, renewable and lightweight building material, wood has always attracted people’s attention^1–5^. However, its intrinsic high flammability brings about fire hazards which tends to cause huge economic losses and casualties^6–9^. Various methods have been developed to improve fire safety of wood, among which, flame-retardant coating is the most striking strategy as it could be easily applicable on wood^10–13^. For example, Pedro et al. proposed a fireproof coating based on lignin, tannins and inorganic nanoparticles, which significantly reduced the linear combustion speed by 50% compared with pure wood^14^. Chu et al. used phosphate ester-polyethylene glycol as fireproof coatings to endow wood with excellent flame retardancy, decreasing the heat release rate (HRR) and total heat release (THR) by 82.4% and 84.3%^15^. However, the materials used are not green enough and the preparation process of the fireproof coating is rather complicated in these works, which limits its widely application, so an easy-to-process fireproof coating method is required.

As a green and easy-to-preparation material, hydrogel, with high content of water and nontoxic features, has be proved an ideal candidate for flame-retardant coating^16–20^. High content water means most heat can be absorbed with the evaporation of water. Nontoxic features ensure no toxic volatiles released during decomposition. These two reasons make hydrogel the outstanding flame-retardant coating material. Hung et al. synthesized a green, tough and highly efficient flame-retardant rigid polyurethane foam (RPUF) based on polyacrylic-polydopamine double network hydrogel through UV-curing^21^. Zhang et al. prepared self-healing, recyclable, and degradable fire-retardant gelatin-based biogel coating via two steps stirring method for green buildings^22^. In our previous work, we have designed a flame-retardant wood based on polyvinyl alcohol/phytic acid (PVA/PA) hydrogel and explored the effect of PA on the flame retardancy and adhesion in PVA hydrogel. Though the hydrogel coating can endow wood with flame retardancy, self-healing ability and high water-retention need to be realized for the targets of long-time use and better flame retardant ability.

Self-healing property can be realized by physical and chemical approaches including diffusion and flow, shape-memory effects, heterogeneous self-healing systems, covalent-bond reformation and reshuffling, hydrogen-bond interaction, Metal coordination bond action and dynamics of supramolecular chemistry or combinations thereof^23–25^. Among these, hydrogen bond is the easiest method to be realized. As a typical dynamic noncovalent bonds, the import of hydrogen bonds endows hydrogel excellent self-healing property without other source and enhance water-retention capacity of hydrogel^26–30^. MXene, a 2D inorganic nanomaterial with plenty of functional groups (–OH, –F and so on) terminally^31–35^, can form many hydrogen bonds with PVA and PA and promote gelation degree of PVA as crosslinking agent to enhance the mechanical properties of hydrogel, which greatly contributes the self-healing and high water-retention properties^36^. Moreover, the high thermal stability and nanosheet structure of MXene can further enhance the flame retardancy of hydrogel. Lin et al. added MXene into chitosan hydrogel coating applied on RPUF, significantly decreased the HRR and THR by 57.2% and 65.5%^37^, showing the excellent flame-retardant property of MXene.

Thus, to address that, a flame-retardant wood based on self-healing, high water-retention and flame retardancy PVA/PA/MXene hydrogel coating was prepared via a facile method in this work. The hydrogel coating was obtained via one-pot method and a freeze–thaw cycle and applied on wood by blading. Tensile test was used to detect the mechanical properties of M-hydrogel, and self-healing and water-retention properties were conducted to see the long service life of hydrogel coating. UL-94, open fire tests and cone calorimetry tests (CCT) were performed to explore the flame retardancy of M-hydrogel-coated woods. The M-hydrogel coating endows wood with outstanding flame retardancy. The property enhancement mechanisms have been given in detail.

## Materials and methods

### Materials

PVA (Mw∼145,000) was obtained from Aladdin Biochemical Technology Co., Ltd. (Shanghai, China). PA solution (50 wt%, in water) was purchased from Sinopharm Chemical Reagent Co., Ltd. LiF (lithium fluoride) was got from Macklin Biochemical Co., Ltd. (Shanghai, China). HCl (hydrochloric acid, 36.0–38.0 wt%, in water) was provide by Guangzhou Chemical Reagent Factory (Guangzhou China). Ca(OH)_2_ (calcium hydroxide, 95.0 wt%) was gained from Fuchen Chemical Reagent CO., Ltd. (Tianjin, China). MAX (Ti_3_Al_2_C_2_) powder (~ mesh 400) was supplied by Kai Xi Ceramic Materials Co., Ltd. (Yantai, China). The materials mentioned above were used as received without further purification.

### Preparation of MXene (Ti_3_C_2_T_X_), MXene hydrogel coating (M-hydrogel) and M-hydrogel-coated wood

MXene nanosheets were synthesized by the etching and delaminating method. The etching solution was prepared by adding 4 g of LiF to 60 ml HCl and 20 ml DI water, followed by stirring for 30 min. Then 4 g MAX was slowly added into the solution and the stirring time lasted about 24 h. The acidic suspension was washed with DI water until a pH value over 5 via centrifugation at 3500 rpm (5 min per cycle) for about 3 times. Next, the DI water was replaced by alcohol and the suspension was ultrasonicated for 2 h and centrifuged at 10,000 rpm for 10 min. Subsequently, the alcohol was replaced by DI water and ultrasonicated for 20 min. Lastly, the supernatant was collected via centrifugation at 3500 rpm for 5 min and freeze-dried to get the MXene powder.

M-hydrogel and M-hydrogel-coated wood was prepared as the following way: First, DI water was added in 15 ml PA until the weight reached 30 g. Then, PVA and MXene powder was mixed in PA solution with one-pot method and the mixture was stirred for 2 h at 95 °C to get the pre-hydrogel. Finally, M-hydrogel was provided after a freeze–thaw cycle of pre-hydrogel and M-hydrogel-coated wood was obtained with pre-hydrogel coated on wood via blading followed by a freeze–thaw cycle. The component details are listed in Table 1.

**Table 1:** The composition of different samples.

Samples	PVA (g)	PA solution (g)	MXene (g)
Pure wood	–	–	–
M0	3.0	30.0	0
M0.1	3.0	30.0	0.1
M0.2	3.0	30.0	0.2
M0.3	3.0	30.0	0.3

### Characterization

The morphology of M-hydrogel-coated wood and uncoated wood was characterized by Scanning Electron Microscope (SEM, Merlin, Germany Zeiss) equipped with energy dispersive X-ray (EDX) system. X-ray Photoelectron Spectroscopy (XPS, Kratos Axis Ulra DLD, Britain) was used to analyze chemical composition of the hydrogel samples. The functional groups of M-hydrogels and MXene were detected by Fourier Transform Infrared Spectrometer (FTIR Spectrometer, VERTEX 70, Bruker German). X-ray diffraction (XRD, D8 ADVANCE, Bruker) was performed to evaluate the crustal structure of MXene. The thermal stability of hydrogel samples was evaluated via thermogravimetric analyzer (TGA, NETZSCH STA 449F3 Germany) with a heating rate of 10 ℃ min^-1^ under N_2_ flow of 60 mL min^−1^ from 30 to 800 °C.

### Mechanical, self-healing and water-retention tests

The stress–strain curves were obtained by a universal electronic tensile machine with a tension speed of 300 mm min^−1^ (Shimadzu, Japan), the size of dumbbell tape hydrogel samples is 30 × 5 × 3 mm^3^. The samples of stress–strain and adhesion test were performed at least five times. Cross-hatching test was performed to evaluate the adhesion of M-hydrogel coating according to ASTM Designation: D 3359–97.

To evaluate the self-healing property of M-hydrogel, the sample was cut into two pieces and put together with the fracture surface contacted. Some water was sprayed on the crack and then the sample placed in the ambient environment overnight. The water-retention test was performed in room environment with temperature at 25 °C and RH at 60%. The swelling property was examined by immersing samples into DI water and weighting every hour.

### Combustion behavior and heat insulation tests

UL-94 (CFZ-2 Analytical Instrument company, Jiangning, China) was used to evaluate the flame retardancy of all samples, the size is 125 × 13 × 3 mm^3^, according to ASTM D 3801. Open fire test was performed for hydrogel-coated wood and uncoated wood by exposing samples (50 × 50 × 3 mm^3^) directly to propane torch flame (≈ 2000 °C) for 20 s. The length of propane torch was controlled about 5 cm and the angle between the sample and flame was fixed at 90°. The CCT test was performed on a cone calorimeter (FTT, UK) at a heat flux of 50 kW m^−2^ with samples dimension of 100 × 100 × 3 mm^3^, according to ISO 5660-1. The distance between the surface of samples and the cone was 60 mm. For heat resistance test, infrared camera was used to record the temperature raising speed of hydrogel-coated and uncoated samples.

## Results and discussion

### Structure characterization

**Figure 1:**
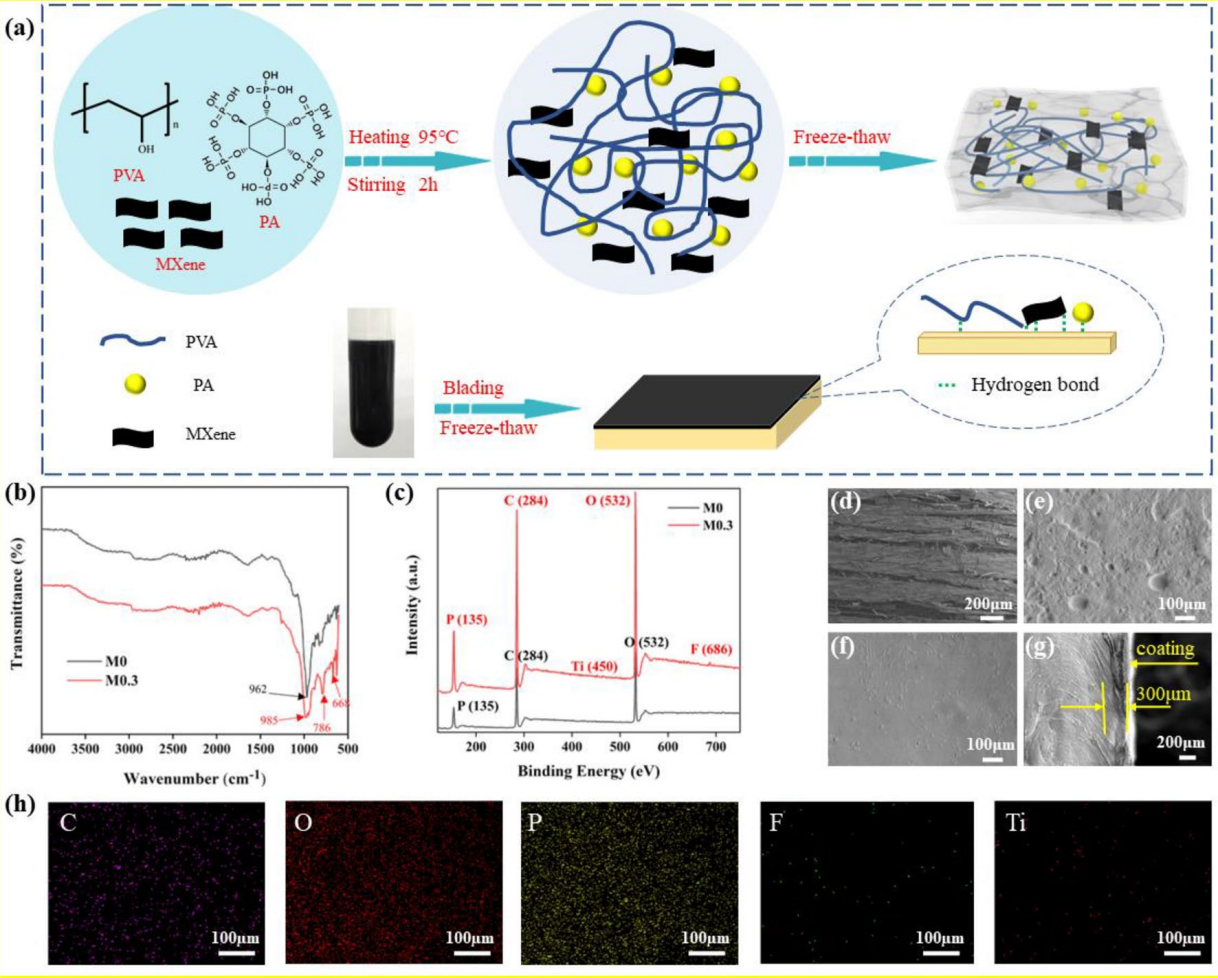
The synthetic process and characterization of M-hydrogel, M-hydrogel-coated and uncoated wood. (**a**) The illustration of hydrogel and hydrogel-coated wood preparation procedures. (**b**) FTIR spectra and (**c**) XPS of M0 and M0.3 hydrogel. SEM image of the surface of (**d**) pure wood, (**e**) M0 hydrogel-coated wood, (**f**) M0.3 hydrogel-coated wood and (**g**) the cross-section structure of M0.3 hydrogel-coated wood. (**h**) EDX of M0.3 hydrogel.

MXene was obtained by etching Al layer from MAX and then delaminated via sonicating (Fig. S1). The diffraction peak at 6.8° in XRD result (Fig. S2) and vibration peaks at 3450 cm^−1^, 1368 cm^−1^ (–OH), 550 cm^−1^ (Ti–O) in FTIR result (Fig. S3) demonstrate the successful preparation of MXene^38^. The synthesis process of M-hydrogel can be divided into two parts: (I) PVA, PA, and MXene were mixed in DI water and the mixture was stirred at 95 °C for 2 h to make pre-hydrogel. (II) Then M-hydrogel could be obtained after the pre-hydrogel frozen in refrigerator overnight and thawed in room temperature. In our previous work, it has been proved PA could enhance mechanical performances of hydrogel by promoting gelation degree^39^. MXene here could make the same contribution as gelation agent which formed more hydrogen bond. To get the M-hydrogel-coated wood, the pre-hydrogel was applied on the surface of wood via blading and the thickness was controlled at 300 µm, then the wood with coating experienced a freeze–thaw cycle to gain M-hydrogel-coated wood (Fig. 1a). The component in hydrogel (PVA, PA, MXene) can formed hydrogen bonds with cellulose of wood, which insure the adhesion between M-hydrogel and wood. All samples are named Mx-hydrogel, in which x represents the content of MXene. The component details of different samples can be seen in Table 1.

The effect on hydrogel with the introduction of MXene was explored by FTIR spectra (Fig. 1b) and XPS (Fig. 1c). It can be seen a distinct characteristic peak in M0 and M0.3 at 962 cm^−1^ and 983 cm^−1^, which should be assigned to vibration peak of PA^40^. Compared with M0, there are new adsorption peaks appeared at 786 cm^−1^ and 668 cm^−1^ in M0.3. These two characteristic peaks may be due to deformation vibrations when the hydroxyl groups (–OH) contained in PVA and PA interact with the functional group of MXene under the formation of hydrogen bond^41^. XPS shows that except for P (135 eV), C (284 eV), O (532 eV), the characteristic elements of Ti (450 eV) and F (686 eV) arise in M0.3 sample resulting from MXene component. Also, as is shown in Fig. 1h, the results of EDX of M0.3 hydrogel confirms that the ingredients are mixed evenly. All tests above prove the M-hydrogel is synthesized successfully. SEM images reveal the morphology of M-hydrogel-coated and uncoated wood. The surface of pure wood is rough and many strip cracks could be seen because of the plenty of wood fibers (Fig. 1d). It is noting that appearance of M-hydrogel-coated wood is smooth, and all cracks are sealed which would make a positive contribution to flame retardancy (Fig. 1e,f). The cross-section image in Fig. 1g shows the coating combined with wood closely at the thickness of 300 µm. It can not only ensure the flame retardancy, but also do no harm to the mechanical property of wood.

### Mechanical and self-healing properties

**Figure 2:**
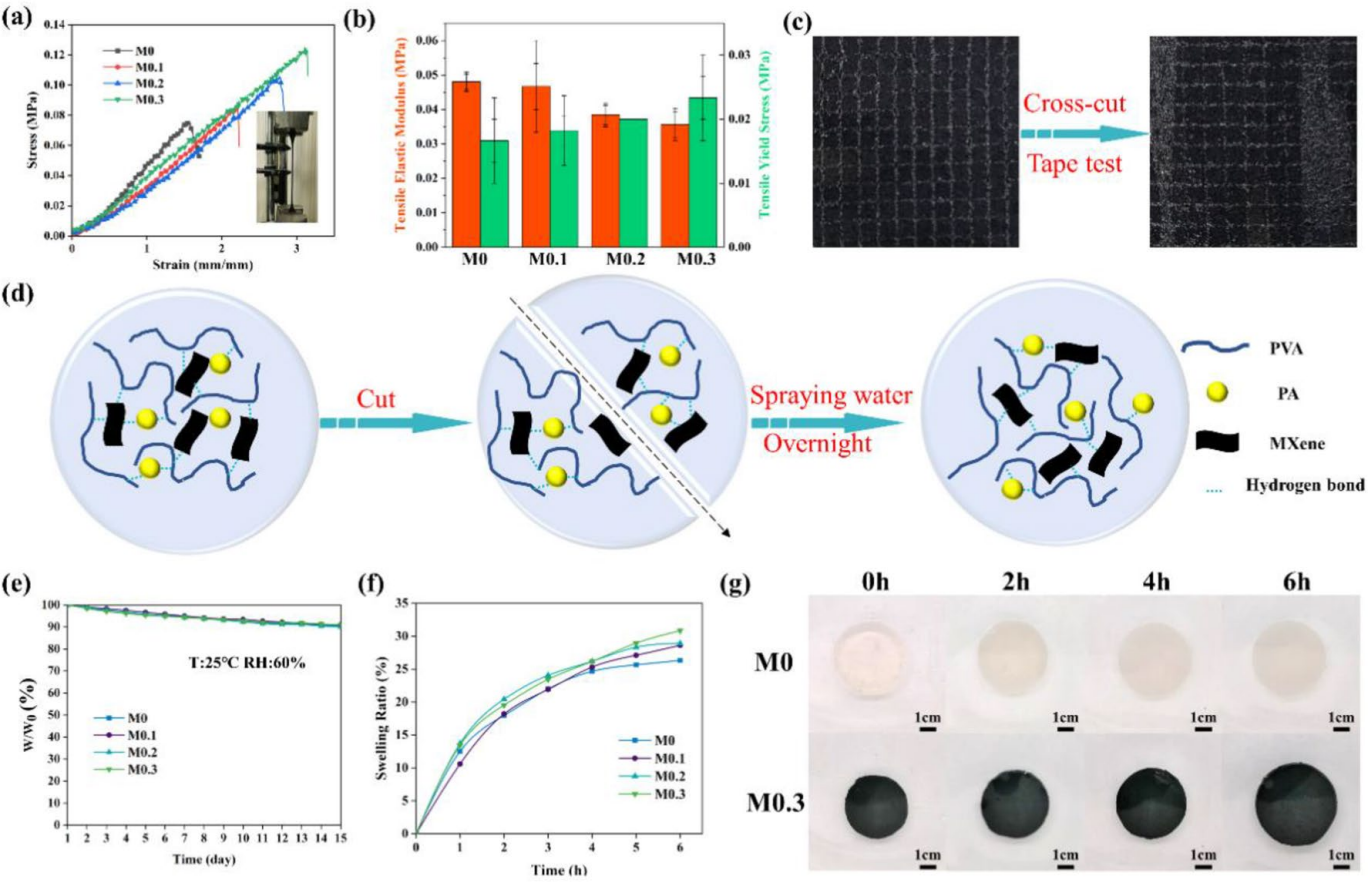
The mechanical properties, self-healing, water-retention, and swelling ratio of M-hydrogel. (**a**) The stress–strain curves of M-hydrogel and the physical photo is inserted. (**b**) Tensile elastic modulus and tensile yield stress of M-hydrogel. (**c**) Cross-hatching test of M0.3 hydrogel-coated wood. (**d**) The schematic illustration of self-healing principle of M-hydrogel. (**e**) Water retention of M-hydrogel, W_0_ and W represent the original and current weight of hydrogels. (**f**) Swelling ratio of M-hydrogel and (**g**) the photographs of M-hydrogel under different immersing time.

The mechanical properties of M-hydrogel were evaluated by tensile tests. As is shown in Fig. 2a, with the higher content of MXene, the stress and strain of M-hydrogel increase significantly. The stress and strain of M0, M0.1, M0.2, M0.3 hydrogel could reach 0.076, 0.084, 0.105, 0.124 MPa and 150%, 220%, 280%, 310%, respectively. The enhancement in stress–strain curves of M-hydrogel can be ascribed to more hydrogen bonds and stronger polymer chain entanglement with the importation of MXene^42^. The functional groups in MXene can form hydrogen bonds with PVA and PA, resulting in more hydrogen bonds in M-hydrogel. Besides, MXene can act as gelation agent in M-hydrogel to heighten the entanglement of PVA chains. The two reasons are the main factors contributing to strengthen of M-hydrogel. The tensile elastic modulus and yield stress of M0, M0.1, M0.2, M0.3 hydrogel are 0.048, 0.047, 0.038, 0.036 MPa and 0.017, 0.018, 0.020, 0.023 MPa (Fig. 2b). Lower tensile elastic and higher tensile yield stress modulus means less stiffness of M-hydrogel and it can endure bigger deformation, which are coherent with the results of stress–strain curves. Furthermore, the M0.3 hydrogel could be twisted, knotted, and resist the weight of 500 g which reflect the excellent mechanical properties of M0.3 hydrogel (Figs. S4, S5). As a flame-retardant coating, adhesion is another important aspect to think over. We performed cross-hatching test to investigate adhesion of M0.3 hydrogel coating on wood. From Fig. 2c, there is evenly none coating falling off after the tape test. The result exhibits wonderful adhesion of M0.3 hydrogel coating on wood.

It should be considered that when crack appears, it would have adverse effect on the flame retardancy as dense coating is essential for fireproofing, so self-healing is important. To explore the self-healing principle of M-hydrogel, we cut a cuboid M0.3 hydrogel into two blocks, then the fracture section was contacted closely, and some water was sprayed. Finally, the two blocks of hydrogel self-healed into a whole hydrogel overnight in ambient environment (Fig. S6). The schematic illustration of M-hydrogel self-healing principle is demonstrated in Fig. 2d. When the hydrogel was cut into two pieces, the hydrogen bonds among hydrogel were destroyed. However, as dynamic and reversible non-covalent bonds, hydrogen bonds could be rebuilt when the fracture section contacted with water spraying, which ensure the self-healing property of M-hydrogel^43^.

### Water retention and swelling ratio

As a fireproof coating, water is crucial for M-hydrogel in flame retardancy. Conventional hydrogels lost its water easily due to evaporation^44^. In our previous work, we have proved that PV could improve the water retention of PVA hydrogel significantly by bring more hydrogen bonds between PVA and PA^45^. Here MXene was added under the same reason. It is gratifying that all M-hydrogel samples holds it weight over 90% after 15 days under the occasion of 25 °C and relative humidity of 60% (Fig. 2e). The comparison of water maintenance of this work with other hydrogel coating was listed (Table S1) to verify the excellent water capacity of PVA/PA/MXene hydrogel. Further experiments were conducted to investigate the water adsorption capacity of M-hydrogels. The results in Fig. 2f exhibit that with the increasing content of MXene, water capacity of M-hydrogel also increases. The swelling ratio of M0, M0.1, M0.2, M0.3 hydrogel can reach 26.16%, 28.39%, 29.16% and 31.08%, respectively. This phenomenon should be assigned to more hydrogen bonds interaction owing to more MXene and the functional groups in MXene (–F, –OH) endows the M0.3 hydrogel stronger water adsorption capacity^46^. The photographs of M0 and M0.3 hydrogel under different water adsorption are listed in Fig. 2g. When immersed in water for the same time, M0.3 hydrogel is bigger than M0 hydrogel. Other M-hydrogel water capacity image are shown in Fig. S7. All M-hydrogels immersed in water exhibit the same phenomenon, which is coincident with results in Fig. 2f.

### Flame retardancy

**Figure 3:**
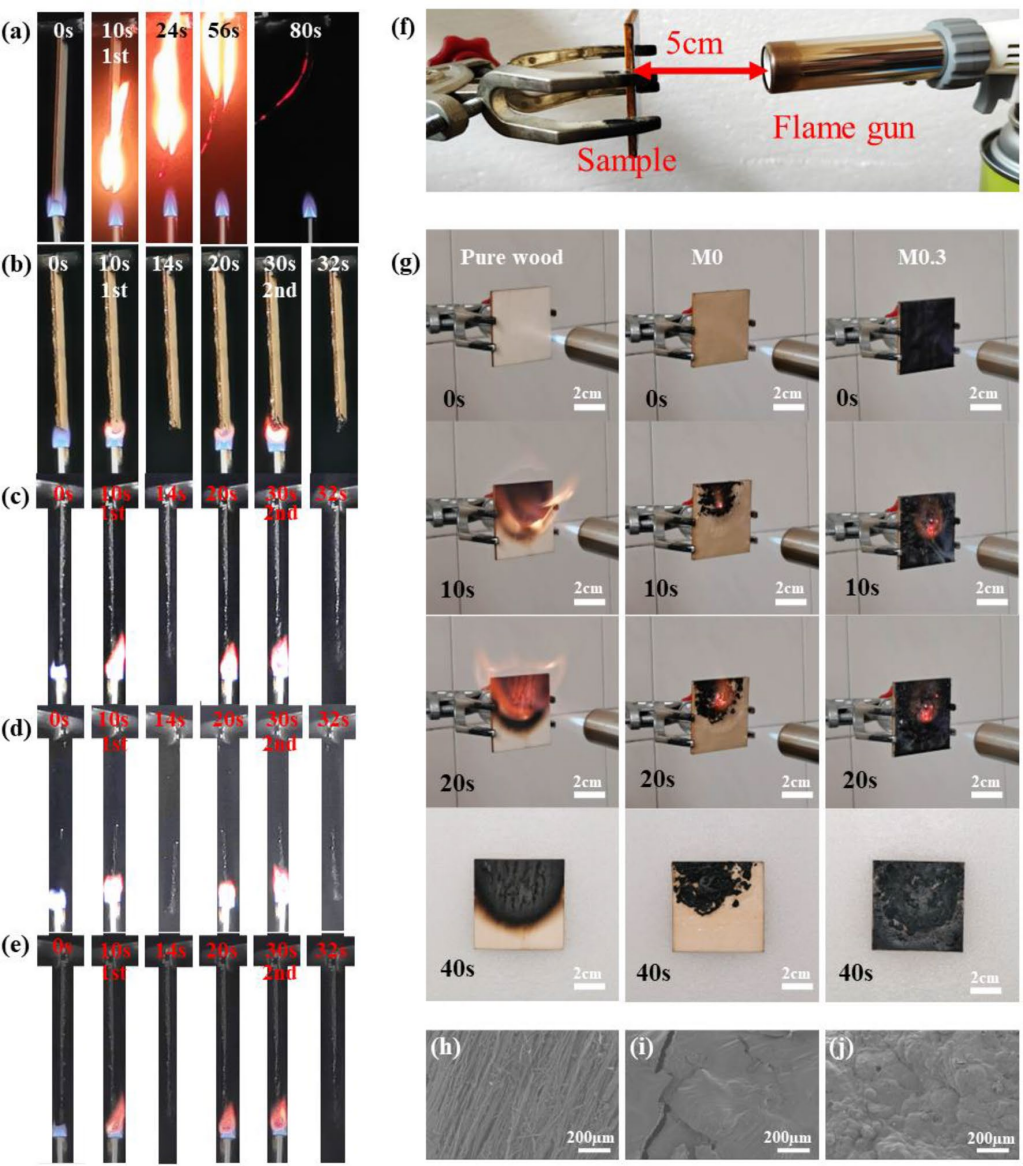
Combustion behavior of M-hydrogel-coated and uncoated samples. The photographs of UL-94 test of (**a**) wood, (**b**) M0, (**c**) M01, (**d**) M0.2 and (**e**) M0.3 hydrogel-coated wood. (**f**) The image of open fire test process. (**g**) The photos of pure wood and M-hydrogel-coated wood in open fire test. (**h**) The SEM images of char layer of (**h**) pure wood, (**i**) M0, and (**j**) M0.3 hydrogel-coated wood after open fire test.

UL-94 tests performed to evaluate the flame-retardant rating of M-hydrogel-coated and uncoated wood and the t_1_, t_2_ is listed in Table 2. During the test, pure wood (Fig. 3a) was ignited after the first 10 s burning and cannot self-distinguished until burn out, showing its intrinsic flammability and fire hazard and the rating should be ranked to V2. Obviously, all M-hydrogel-coated wood did not be ignited even after the second burn for 10 s as shown in Fig. 3b–e, which demonstrate the excellent flame retardancy of M-hydrogel coatings. The photos of M-hydrogel-coated and uncoated wood after UL-94 test can be seen in Fig. S8. The related data of all M-hydrogel-coated wood in t_1_ and t_2_ are 0 s listed in Table 2, stating all M-hydrogel-coated wood can reach V0 rating.

**Table 2:** The related data of UL-94 and CCT.

Sample	t_1_ (s)	t_2_ (s)	Rating	TTI (s)	T_pHRR_ (s)	HRR (kW/m^2^)	THR (MJ/m^2^)
Pure wood	–	–	V2	32	70	109.25	23.96
M0	0	0	V0	64	75	64.15	16.41
M0.1	0	0	V0	74	90	56.54	14.65
M0.2	0	0	V0	66	95	58.36	14.72
M0.3	0	0	V0	69	95	63.80	15.30

*t*_*1*_*, t*_*2*_ the first and second after-flame time during UL-94 tests, *T*_*pHRR*_ the time of the appearance of pHRR.

Open fire tests were conducted to see the effect of fireproof coating intuitively. The samples fixed in clamp were controlled at 5 cm distance from the flame gun (Fig. 3f). In Fig. 3g, after 20 s ignition, there is more intense flame on the surface of pure wood compared with M-hydrogel-coated wood (M0.1 and M0.2 hydrogel -coated samples can be seen in Fig. S9). It is because when the temperature reaches a certain point, the internal moisture of wood evaporates completely, then the surface material begins to gradually decompose carbonized, producing combustible gases and forming a stable flame combustion. But the M-hydrogel coatings contain more water which could effectively delay the process and no damage can be seen from M0 and M0.3 hydrogel-coated wood. Also, the char layer formed by hydrogel coatings are denser than pure wood at 40 s. The SEM images are applied to further evaluate the structure of char layer. It is clearly that the char layer of pure wood (Fig. 3h) is loose and demonstrate fibrous dispersion showing poor heat resistance ability. Significantly, the char layer of M0 hydrogel coating (Fig. 3i) is dense and smooth with sheet layer structure. Though cracks still can be seen, it shows a better heat and O_2_ resistance capability. As for M0.3 hydrogel-coated wood, the char layer is the densest and expansion structure appeared, exhibiting the best heat and O_2_ resistance effect in open tests and best fireproof function (Fig. 3j). This is because lamellar structure of MXene can act as good physical barrier and the char layer is faveolate which shows the best heat resistance capacity among the samples. Moreover, the M-hydrogel fireproof coating could be extended application. When used on RPUF, it exhibits excellent flame retardancy, too (Fig. S10).

**Figure 4:**
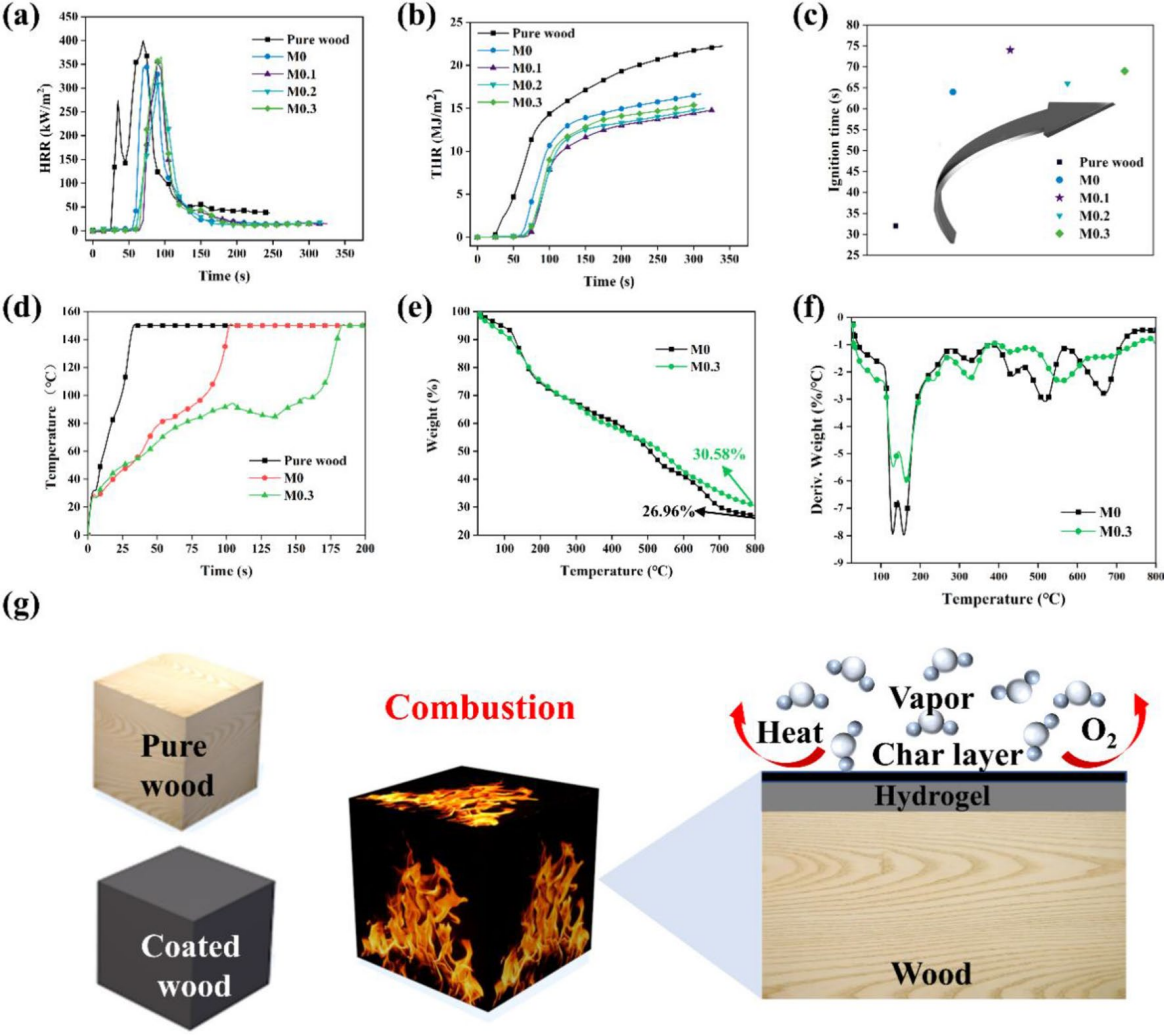
Thermal stability and fire-retardant mechanism of M-hydrogel coatings. CCT results of M-hydrogel-coated and uncoated wood. (**a**) HRR, (**b**) THR, (**c**) TTI. (**d**) Heat- transfer results of pure wood, M0 and M0.3 hydrogel-coated wood. (**e**, **f**) TG and DTG curves of M0 and M0.3 hydrogels. (**g**) The schematic illustration of flame-retardant mechanisms of M-hydrogel coatings.

CCT test was performed to further evaluate the fire safety of M-hydrogel coating and the main parameters are listed in Table 2. According to the results, the time to ignition (TTI) of pure wood is 32 s while M-hydrogel coated wood of M0, M0.1, M0.2 and M0.3 are prolonged to 64 s, 74 s, 66 s and 69 s, respectively (Fig. 4c). The results show the function of water contained in hydrogel coatings to limit the fire spread from surface to inside of wood. It is further proofed in results of heat release rate (HRR) and total heat release (THR) in Fig. 4a,b. There are two peaks in HRR curves of pure wood. When the temperature reaches about 250 °C, the combustion gases of H_2_ and CH_4_ generated along with carbonization of wood surface. These gases are ignited at ignition temperature under heat resource and then fire appears inside resulting in the first peak. When the fire spread to surface of the char layer, no combustion gases decomposed from wood and fuel is applied from volatile matter inside wood. At this circumstance, burning flame changes into flameless combustion and the second peak appears. Compared with pure wood, there are also two peaks in HRR curves of M0 hydrogel-coated wood, but the appearance time of the two peaks is later than pure wood. It is due to M0 hydrogel coating could insulate O_2_ from contact with wood under heating condition. It is worth noting that there is only one peak appears in HRR curves of M0.1, M0.2 and M0.3 hydrogel-coated wood. This may be attributed to the denser char layer formed with introduction of MXene which significantly increases the heat resistance ability of hydrogel coating, resulting in the burning flame and flameless combustion phenomenon occurs at the same time. Besides, the appearance time of peak heat release rate (pHRR) and total heat release (THR) of pure wood and M0, M0.1, M0.2 and M0.3 hydrogel-coated wood are 70, 75, 90, 95, 95 s and 23.96, 16.41, 14.65, 14.72, 15.30 MJ/m^2^, respectively. The results exhibit better fireproof ability of hydrogel coating with the introduction of MXene. It worth noting that with the increase content of MXene, there are slight increase in HRR and THR. It maybe because more MXene bring more C element which results in the increase in HRR and THR. But the little increase can be ignored as it still lower than M0 hydrogel-coated wood in HRR and THR. To compare the flame-retardancy performance of this work with other work, we listed a table to show the excellent fireproof capacity of the M-hydrogel coating in the field of flame-retardant wood (Table S2).

### Thermal degradation behaviors and flame-retardant mechanism

As a critical property, the heat resistance ability of M-hydrogel is evaluated by infrared camera. The M-hydrogel-coated side of the samples is exposed to an open fire, while the temperature of back-side is recorded by an infrared camera and the results can be seen in Fig. 4d. For pure wood, the temperature of back-side rises to 150 ℃ quickly while M0 and M0.3 hydrogel-coated wood show an excellent heat resistance capacitation. At about 100 °C, the temperature rising of M0 and M0.3 hydrogel-coated wood appears a significant postpone. This is because the water evaporation in hydrogel coating brings most heat. In addition, the temperature of M0.3 hydrogel-coated wood stays under 100 °C from 100 to 175 s, which can be assigned to the denser char layer formed by M0.3 hydrogel coating resisting more heat.

Thermogravimetric analysis (TGA) and differential thermal gravity (DTG) results are supplied to explore the thermal property in Fig. 4e,f and the related data are listed in Table 3. T_10%_ appears at 130.56 and 125.86 °C of M0 and M0.3 hydrogels. The decomposition process of M0 and M0.3 hydrogel can be divided into 5 parts. T_I_ and T_II_ at 130.12, 162.25 and 130.68, 158.33 °C of M0 and M0.3 hydrogel can be ascribed to the dehydration of hydroxyl of PVA and PA. T_III_ and T_IV_ at 310.28, 435.42 and 258.35, 327.06 °C of M0 and M0.3 hydrogel can be assigned to the decomposition of side chain and main chain of PVA. T_V_ arises at 644.41 and 678.45 °C of M0 and M0.3 hydrogel can be explained as the decomposition process of phytate groups in PA^47^. When temperature reaches 800 °C, the mass left of M0 and M0.3 hydrogel is 26.96% and 30.58%, which demonstrates the enhancement of thermal stability in hydrogel with the introduction of MXene.

**Table 3:** TGA results of M0 and M0.3 hydrogel.

Samples	T_10%_ (°C)	T_I_ (°C)	T_II_ (°C)	T_III_ (°C)	T_IV_ (°C)	T_V_ (°C)
M0	130.56	130.12	162.25	310.28	435.42	644.41
M0.3	125.86	130.68	158.33	258.35	327.06	678.45

*T*_*10%*_ the temperature of the original mass loss reaches to 10%. *T*_*I*_*, T*_*II*_*, T*_*III*_*, T*_*IV*_*, T*_*V*_ the temperature of the first, second, third, fourth, fifth peaks appear.

Based on all results of the experiments above, the flame-retardant mechanisms can be attributed as following (Fig. 4g): (I) Cooling effect due to the large heat capacity and enthalpy of water evaporation. (II) PA as a phosphorus-based flame retardant, promotes the dehydration of wood into carbon and prolong the red-hot combustion process of wood. (III) Denser and harder char layer due to the introduction of MXene, which insulates most O_2_ and heat spread.

## Conclusion

A PVA/PA/MXene hydrogel fireproof coating was developed and applied on wood via facile one-pot heating and freeze–thaw cycle method. The coating demonstrates excellent self-healing property (without other source) and high water-retention (water content ≥ 90%) due to the increased hydrogen bonds with the introduction of MXene. Besides, the PVA/PA/MXene hydrogel-coated wood shows improved flame retardancy such as reached V0 rating in UL-94 test, increasing TTI (from 32 to 69 s), and decreased HRR and THR by 41.6% and 31.64% in CCT tests, respectively. Multiple flame-retardant mechanisms of (I): cooling effect and large thermal capacity of high content of water within hydrogel; (II) the promotion of char layer formation due to PA (phosphorus-based flame retardant); (III) the compact char layer formed during combustion due to MXene enhances the physical barrier effect for flammable gas, heat and oxygen, the flame-retardant mechanisms above ensures the fire safety of wood. Furthermore, the fireproof coating could be applied on other engineering materials (RPUF), showing its wide application in structure materials with the requirement of fire safety.

## Supplementary Information

Supplementary Information 1.

Supplementary Information 2.

Supplementary Information 3.

## Data Availability

The datasets used and/or analysed during the current study available from the corresponding author on reasonable request.
