# Supplementary material for: Facile fabrication of a novel self-healing and flame-retardant hydrogel/MXene coating for wood
Source: Sci Rep. 2023 Feb 1;13:1826. doi: 10.1038/s41598-023-28228-5 (PMC9892570; doi:10.1038/s41598-023-28228-5)
Supplement: Supplementary file 1 — Supplementary Information 1. [file 41598_2023_28228_MOESM1_ESM.docx]

The datasets used and/or analysed during the current study available from the corresponding author on reasonable request
