# Supplementary figures and images for: Facile fabrication of a novel self-healing and flame-retardant hydrogel/MXene coating for wood
Source: Sci Rep. 2023 Feb 1;13:1826. doi: 10.1038/s41598-023-28228-5 (PMC9892570; doi:10.1038/s41598-023-28228-5)

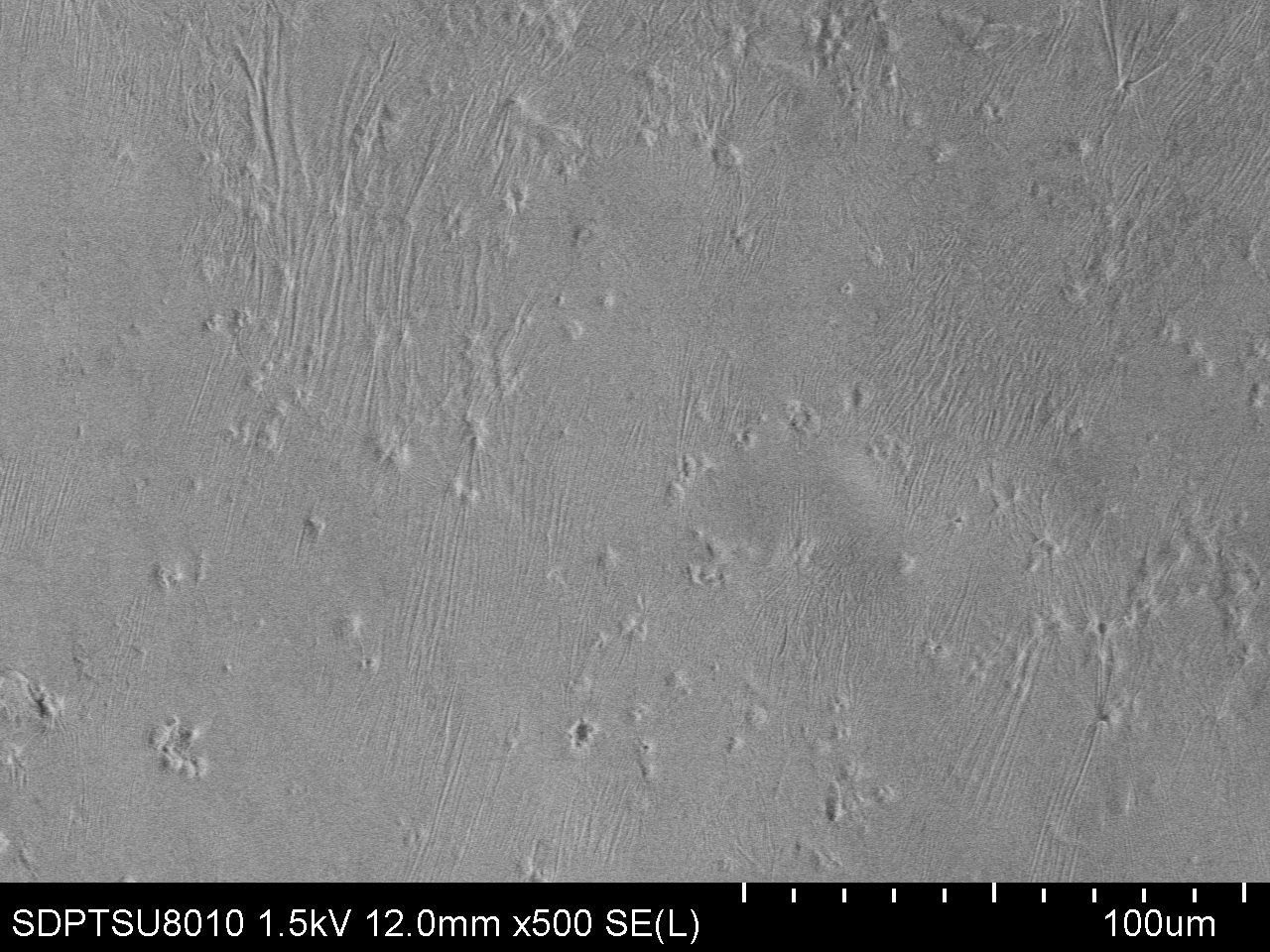

Supplement: Supplementary file 2 — Supplementary Information 2. [file 41598_2023_28228_MOESM2_ESM.zip › SEM-EDX/0.3g.tif]

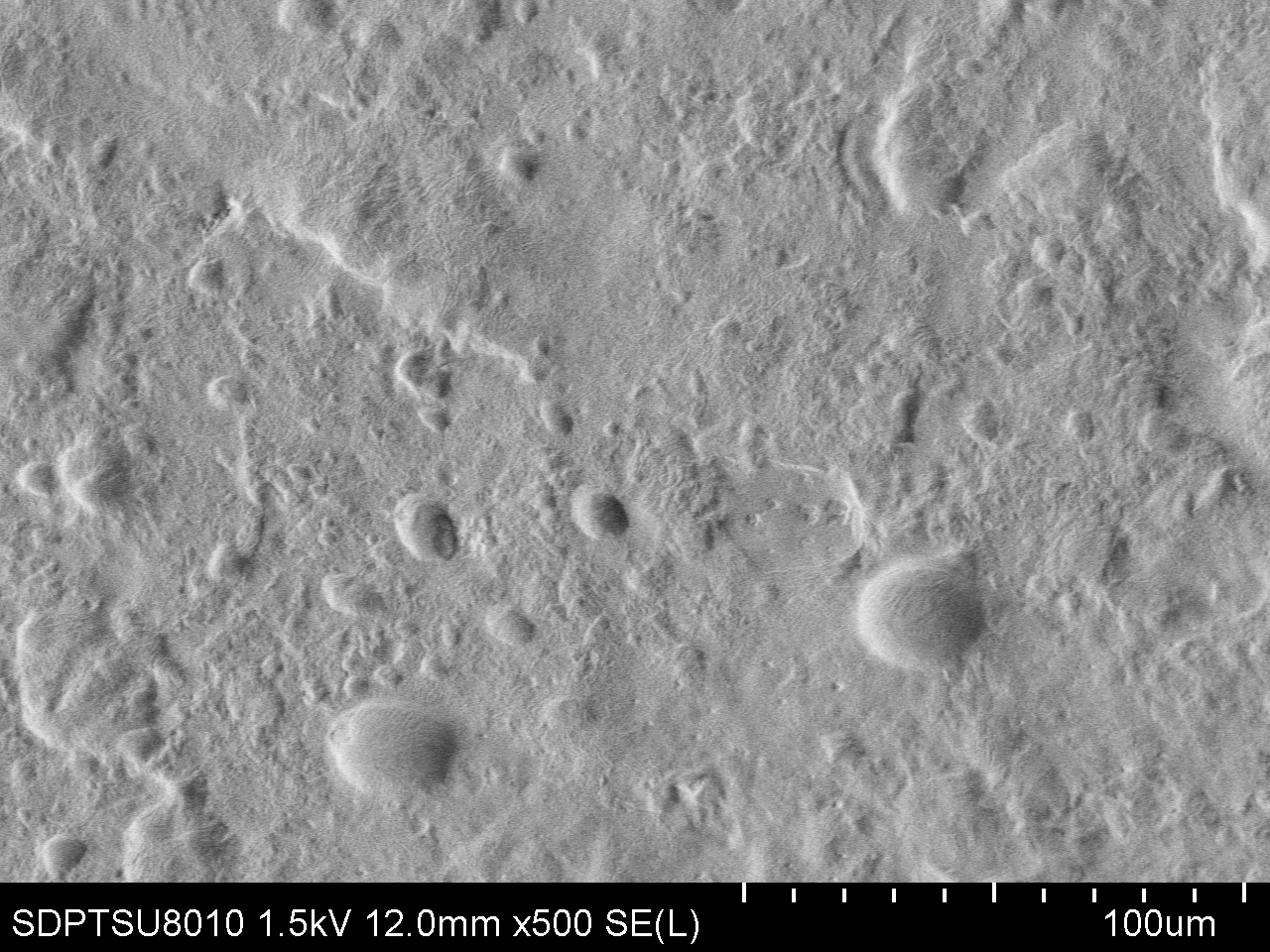

Supplement: Supplementary file 2 — Supplementary Information 2. [file 41598_2023_28228_MOESM2_ESM.zip › SEM-EDX/0g.tif]

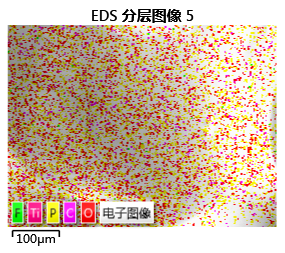

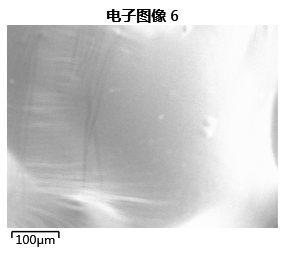

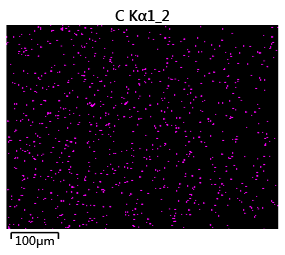

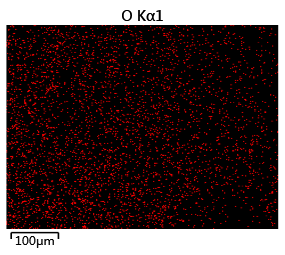

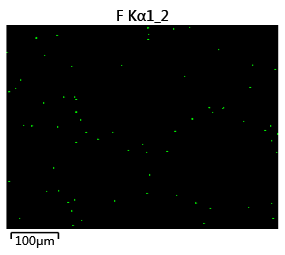

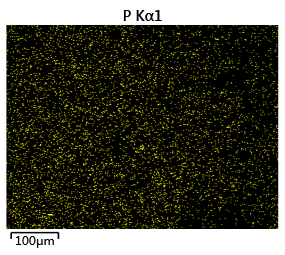

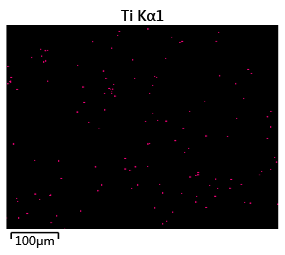

Supplement: Supplementary file 2 — Supplementary Information 2. [file 41598_2023_28228_MOESM2_ESM.zip › SEM-EDX/EDX.docx]

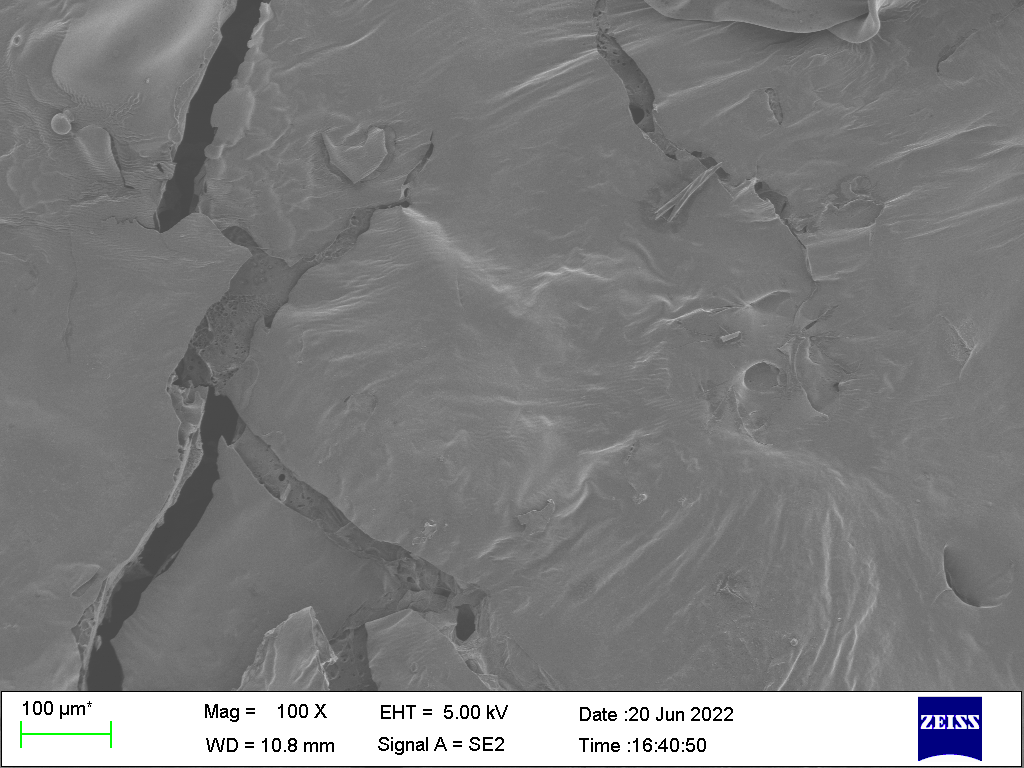

Supplement: Supplementary file 2 — Supplementary Information 2. [file 41598_2023_28228_MOESM2_ESM.zip › SEM-EDX/M0 fired.tif]

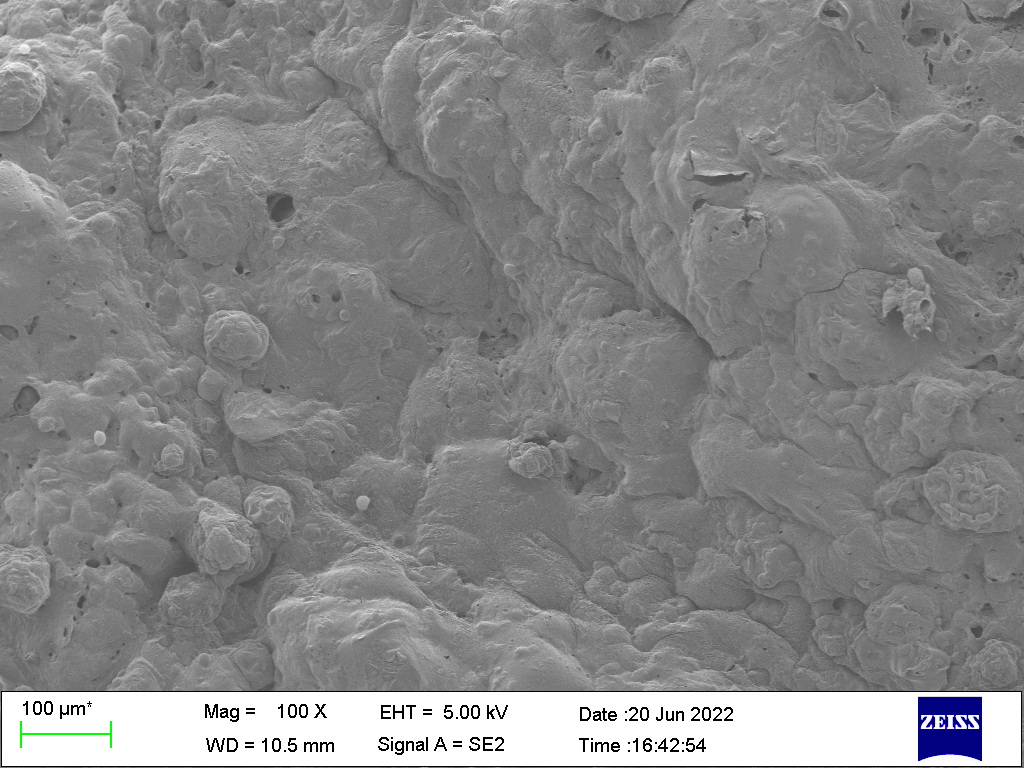

Supplement: Supplementary file 2 — Supplementary Information 2. [file 41598_2023_28228_MOESM2_ESM.zip › SEM-EDX/M0.3 fired.tif]

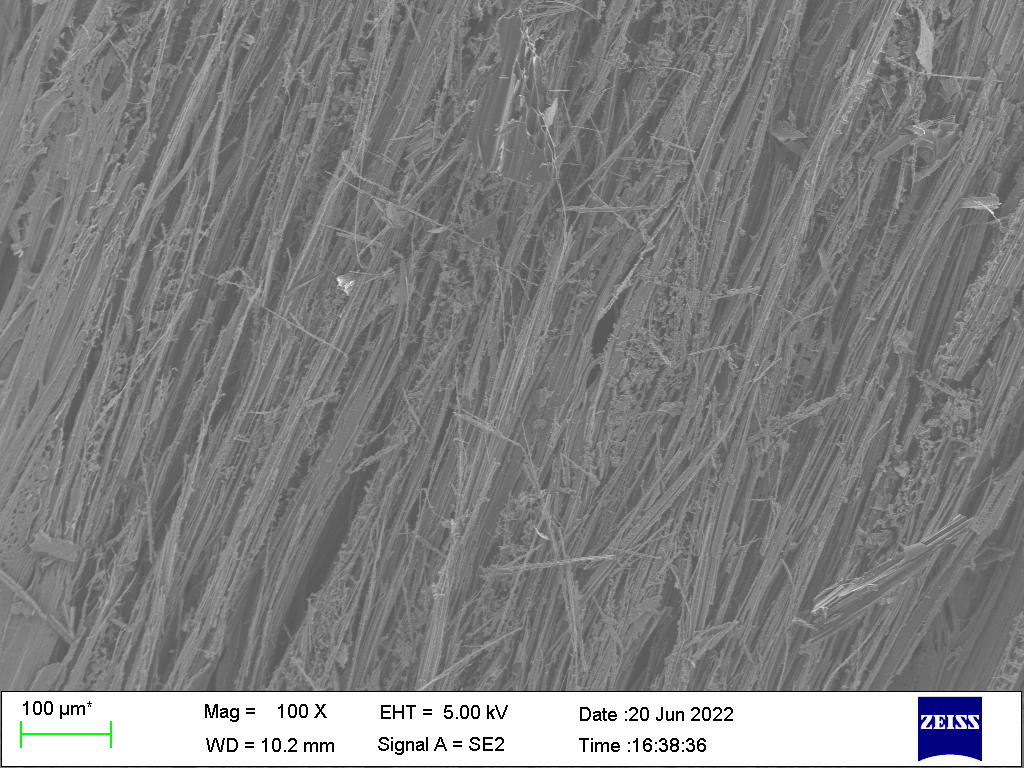

Supplement: Supplementary file 2 — Supplementary Information 2. [file 41598_2023_28228_MOESM2_ESM.zip › SEM-EDX/pure wood fired.tif]
