# Supplementary material for: Facile fabrication of a novel self-healing and flame-retardant hydrogel/MXene coating for wood
Source: Sci Rep. 2023 Feb 1;13:1826. doi: 10.1038/s41598-023-28228-5 (PMC9892570; doi:10.1038/s41598-023-28228-5)
Supplement: Supplementary file 3 — Supplementary Information 3. [file 41598_2023_28228_MOESM3_ESM.docx]

**Facile fabrication of a novel self-healing and flame-retardant Hydrogel/MXene coating for wood**

Xiaojiong Zhao^a,b^, Min Tian^a^, Ruichao Wei^d,e, *^, Saihua Jiang^a, b, c, *^

^a^ Institute of Safety Science and Engineering, School of Mechanical and Automotive Engineering, South China University of Technology, Wushan Road 381, Guangzhou, 510641, P. R. China

^b^ Guangdong Provincial Key Laboratory of Technique and Equipment for Macromolecular Advanced Manufacturing, South China University of Technology, Guangzhou, 510641, P. R. China

^c^ State Key Laboratory of Fire Science, University of Science and Technology of China, Jinzhai Road 96, Hefei, 230026, P. R. China

^d^ Research Institute of New Energy Vehicle Technology, Shenzhen Polytechnic, Shenzhen,

Guangdong 518055, P. R. China

^e^ School of Automobile and Transportation, Shenzhen Polytechnic, Shenzhen, Guangdong

518055, P. R. China

**Corresponding author:**

*E-mail: meshjiang@scut.edu.cn (S.H. Jiang)

*E-mail: rcwei@mail.ustc.edu.cn


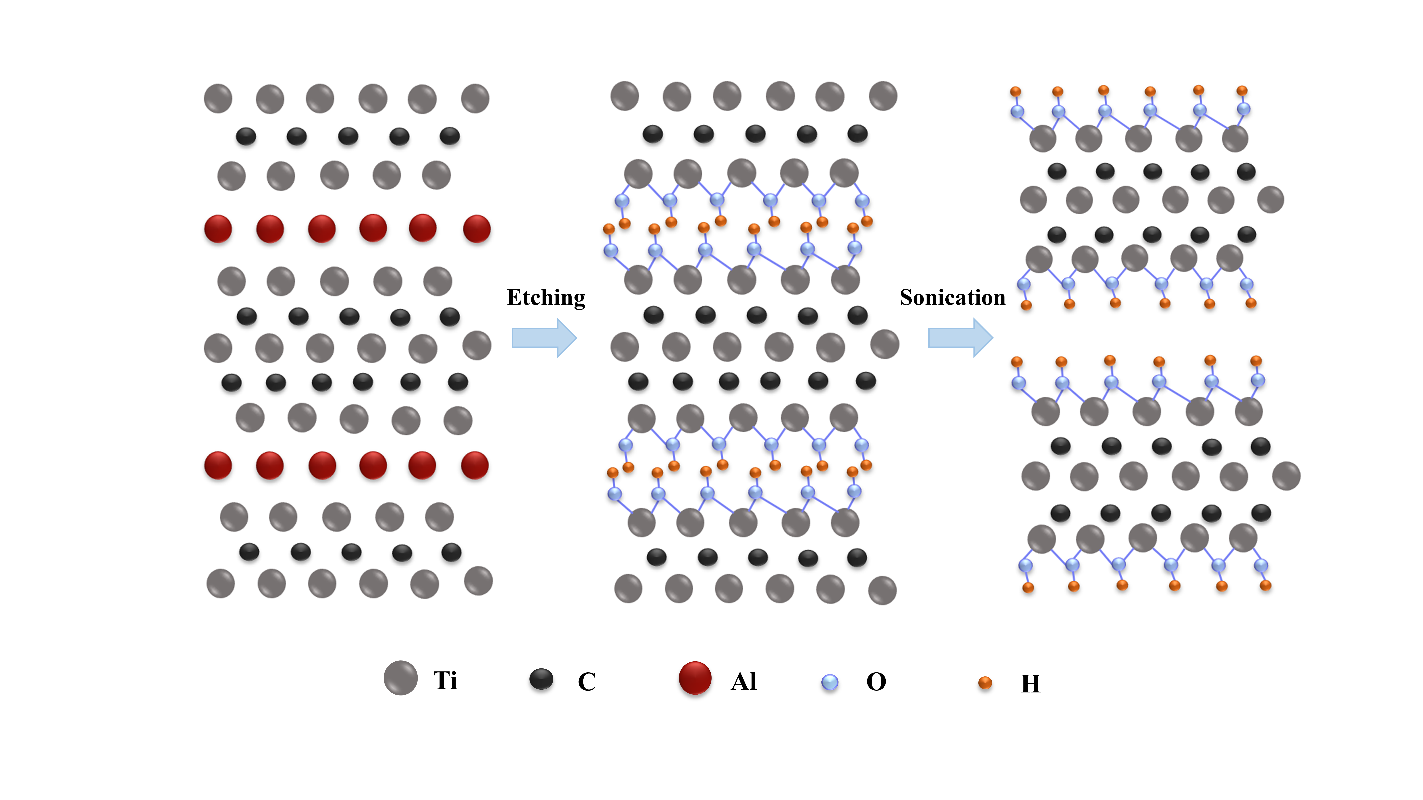


Figure S1. The illustration of the synthetic process of MXene.


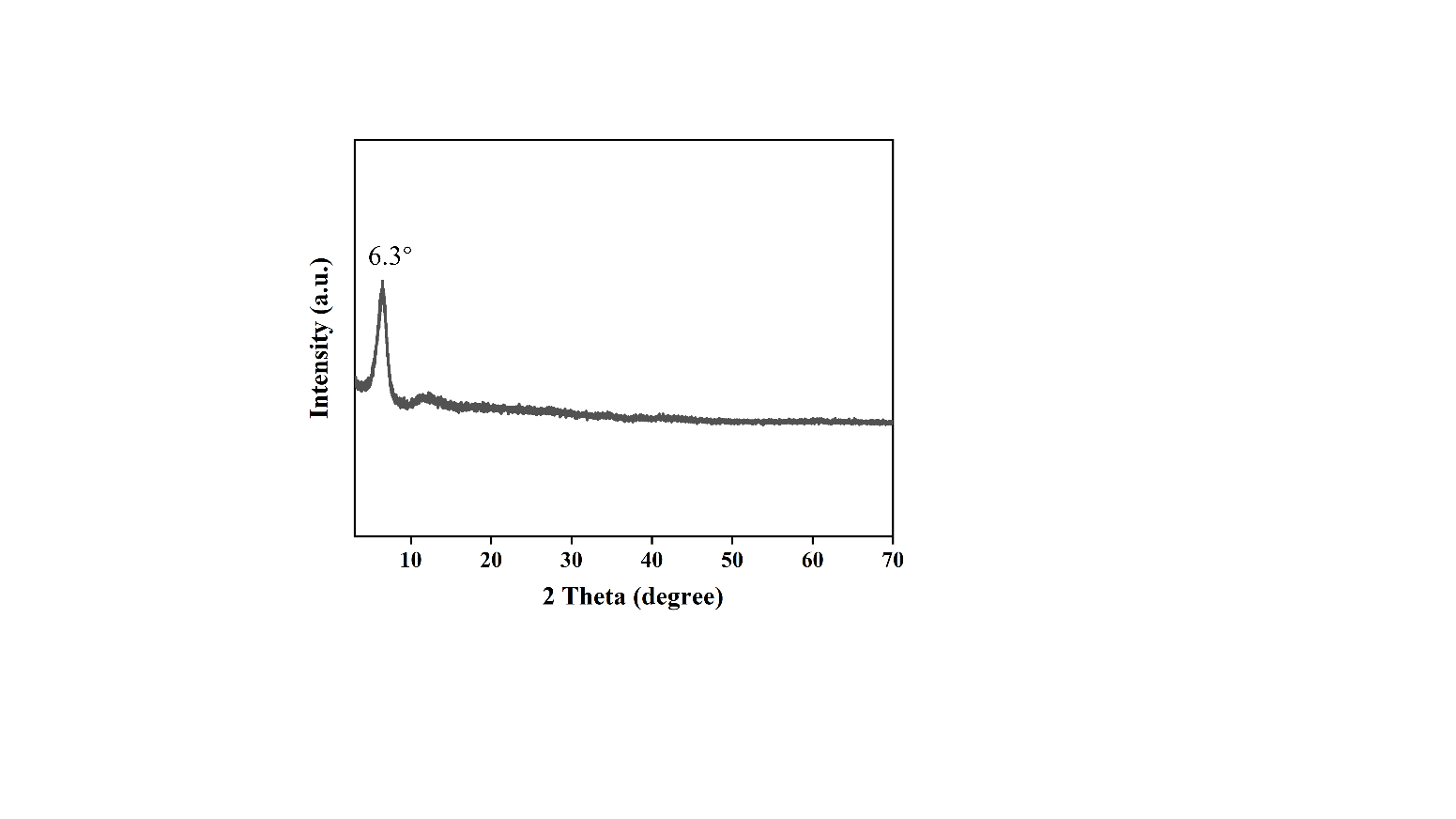


Figure S2. The XRD result of MXene.


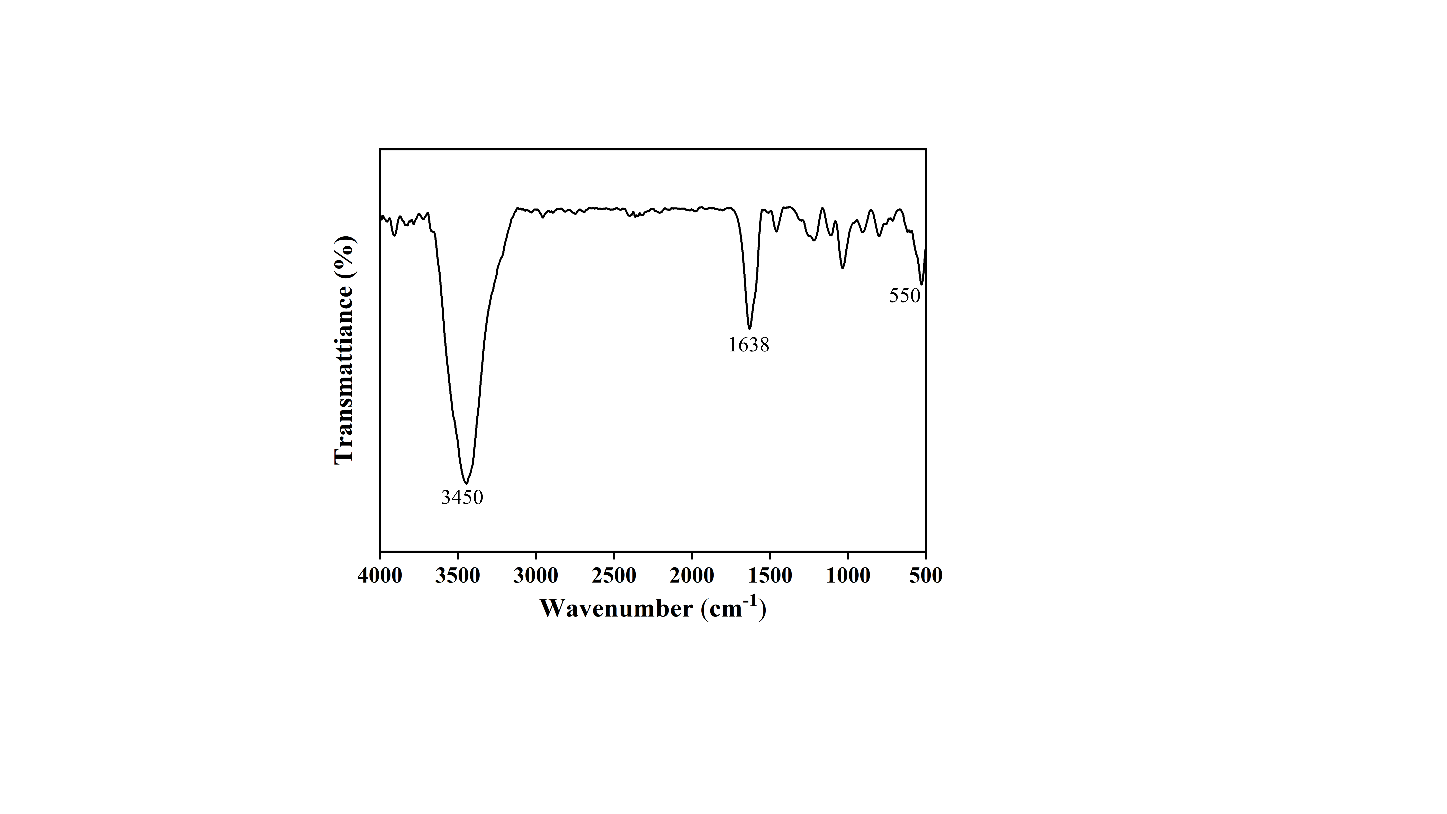


Figure S3. The FTIR result of MXene.


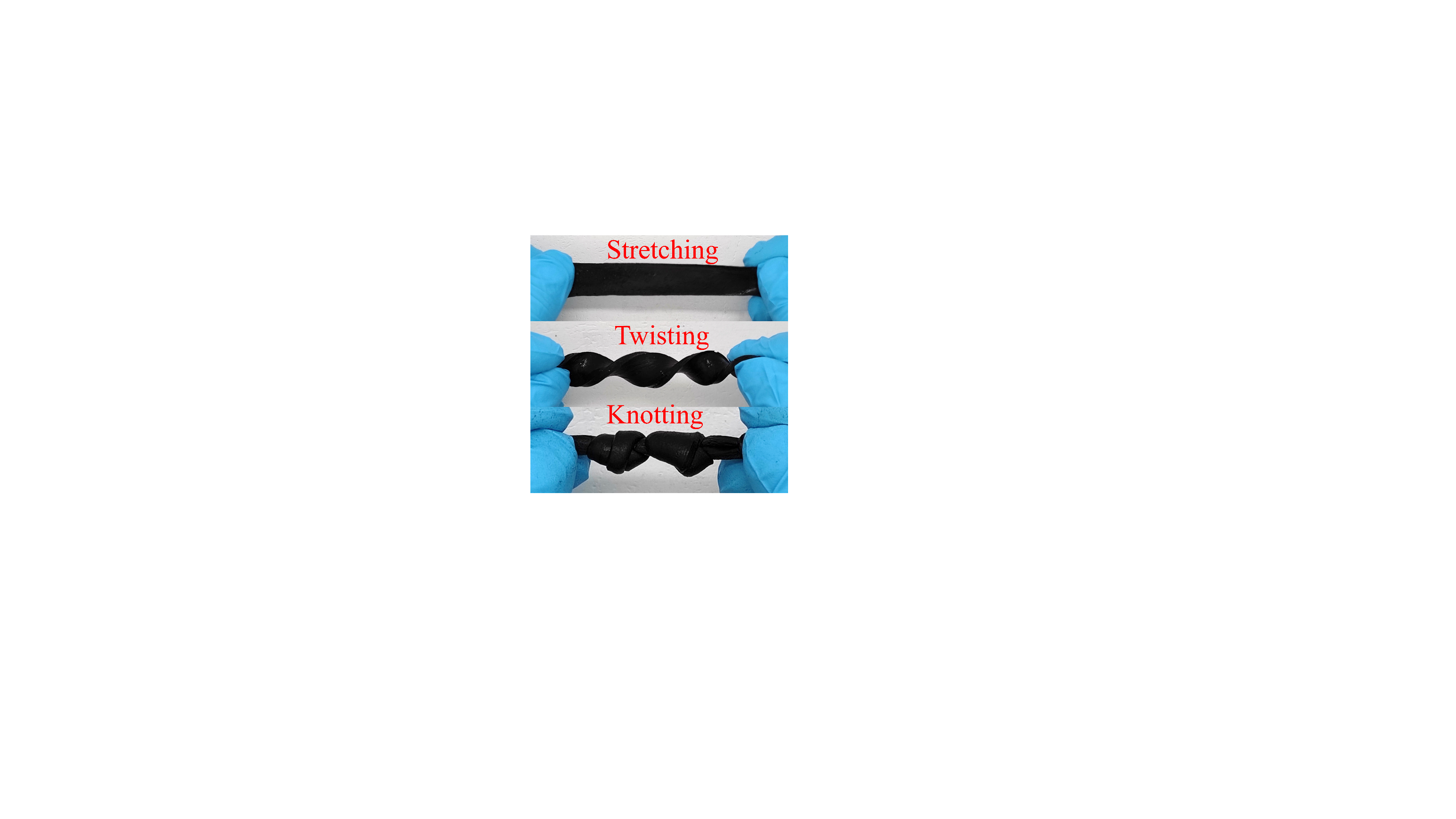


Figure S4. The images of stretching, twisting, knotting of M0.3 hydrogel.


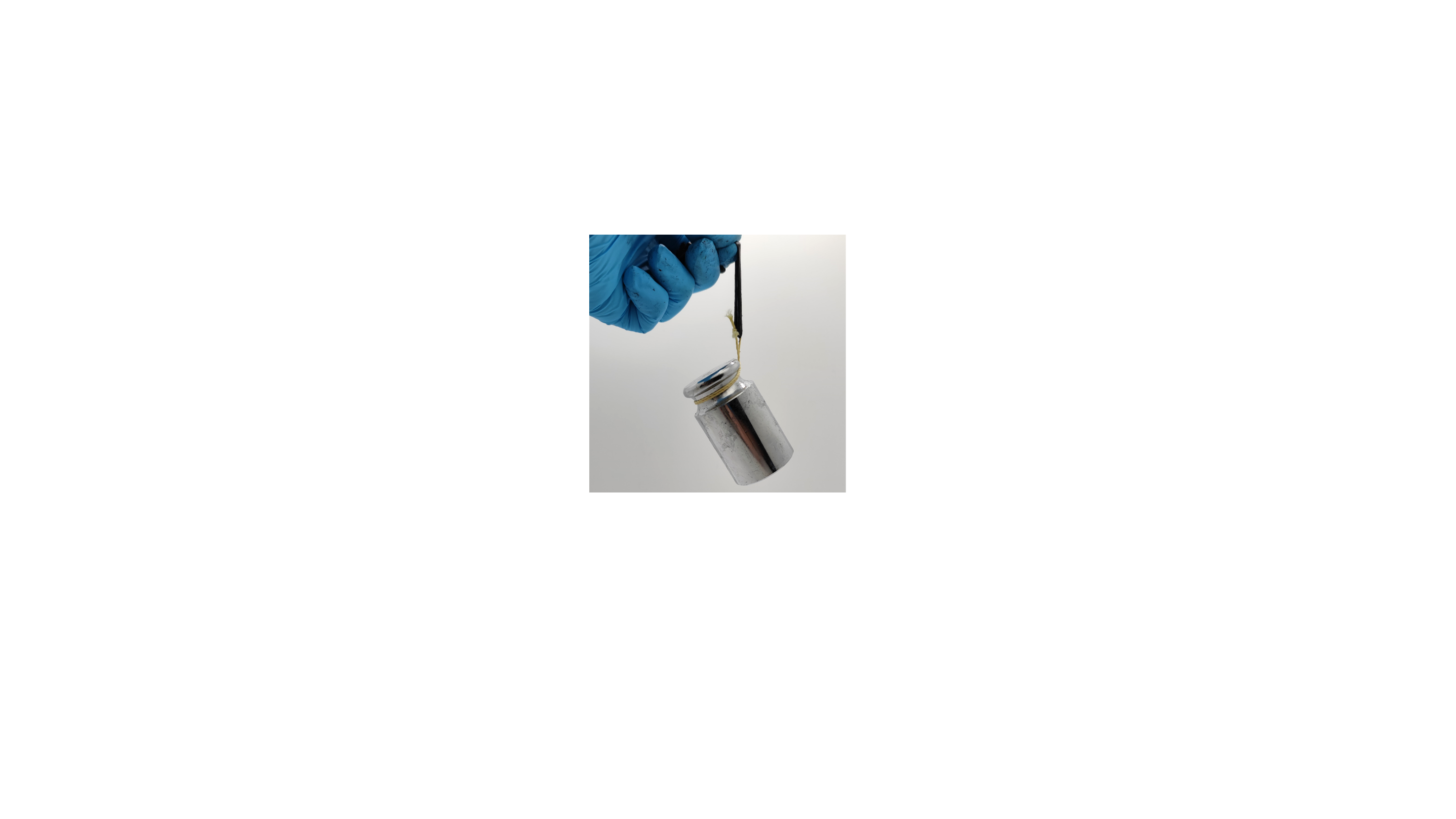


Figure S5. The images of weightlifting of M0.3 hydrogel.


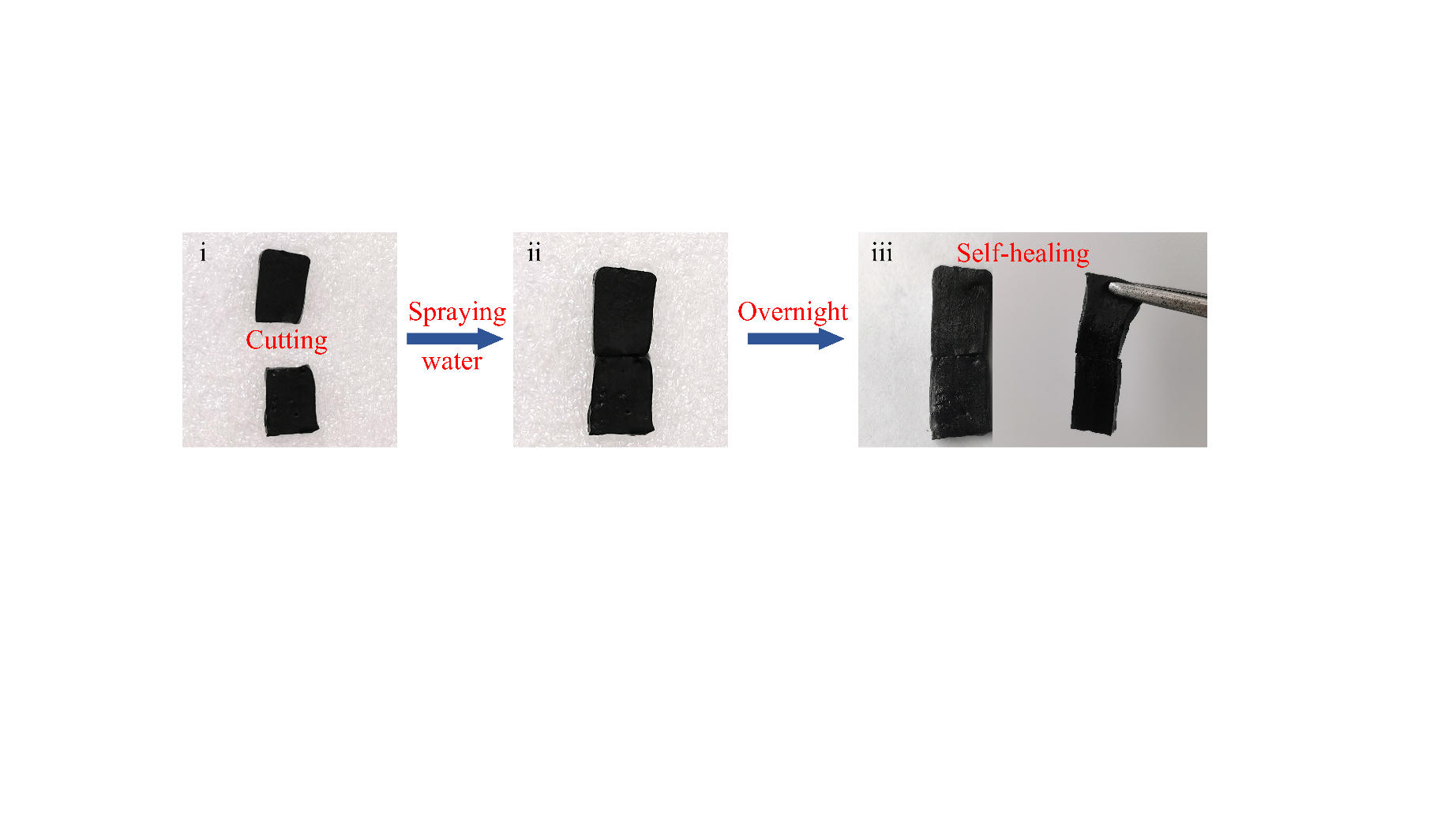


Figure S6. The self-healing process of M0.3 hydrogel.


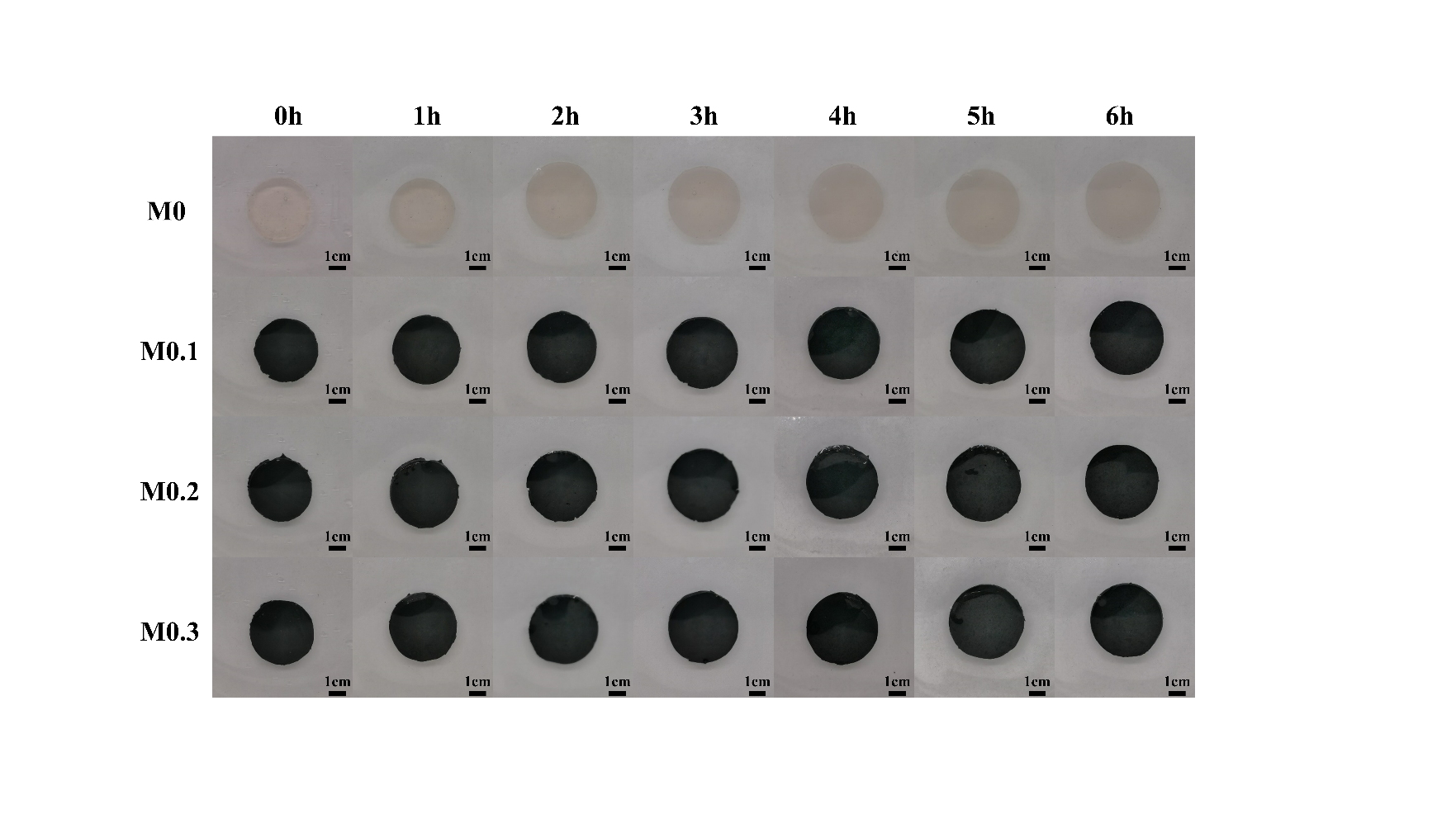


Figure S7. The photographs of M-hydrogels after immersing in water.


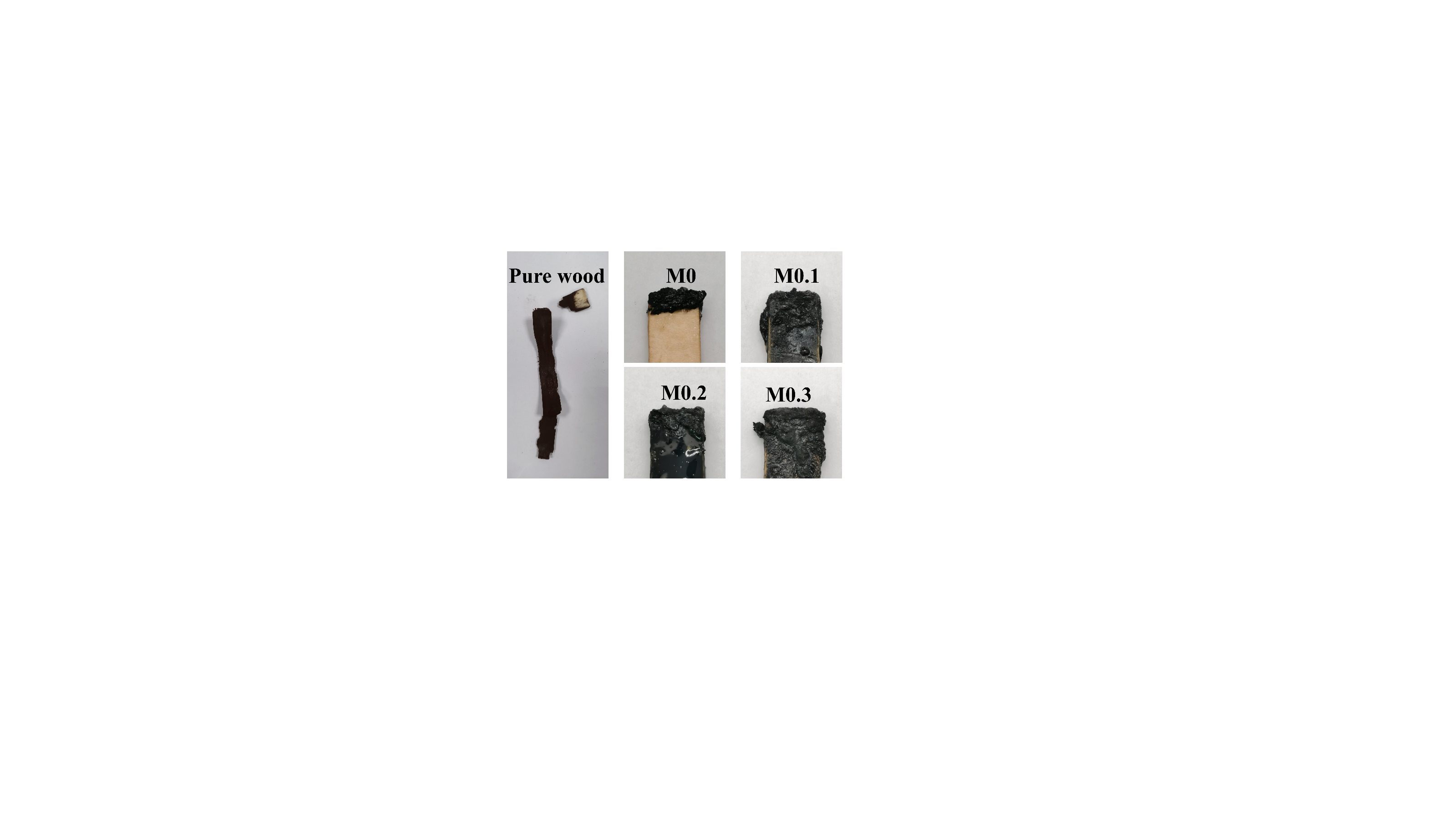


Figure S8. The photographs of M-hydrogels-coated and uncoated wood after UL-94 test.


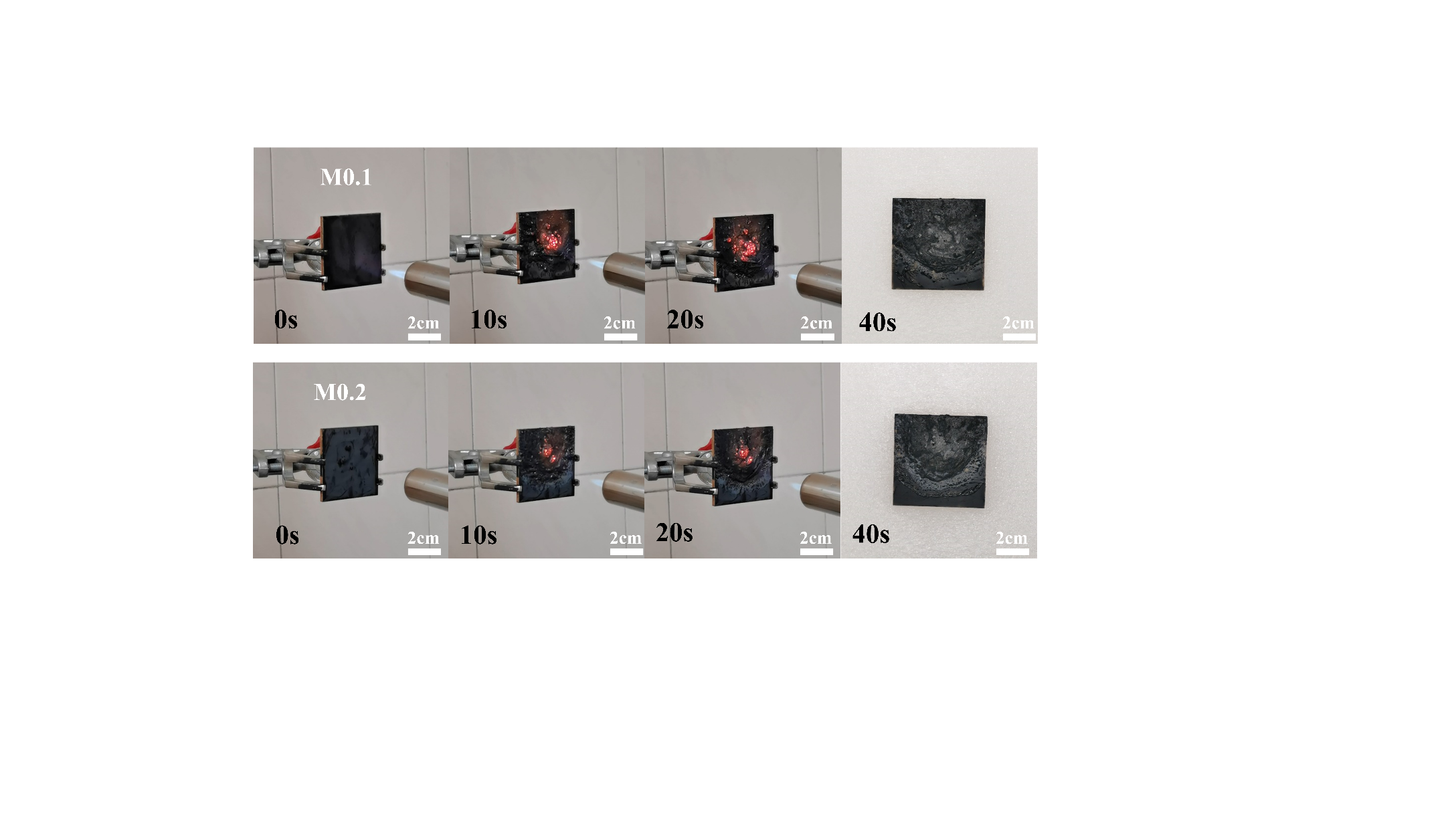


Figure S9. The photos of M0.1, M0.2 hydrogel-coated wood in open fire test.


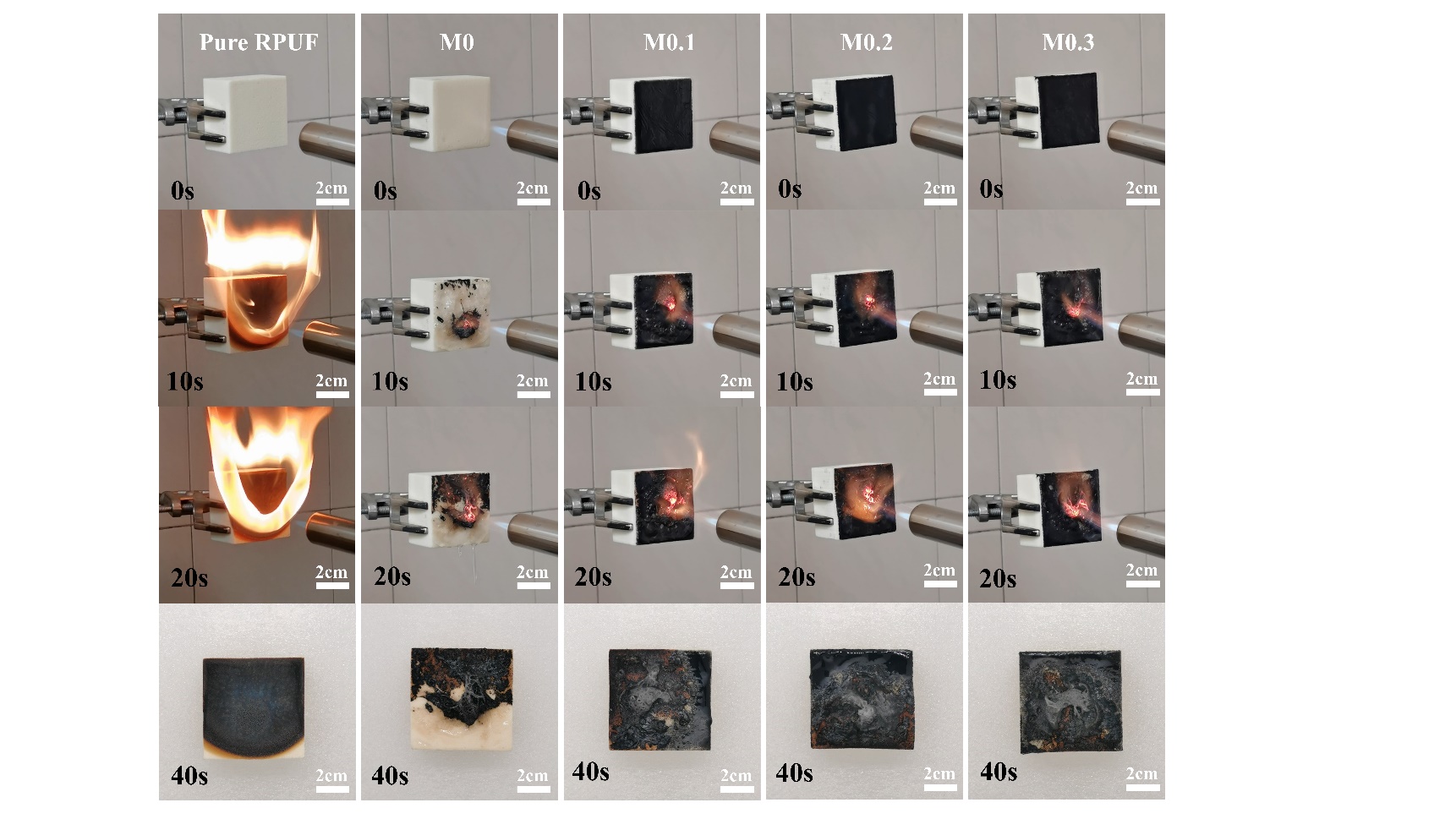


Figure 10. The photos of pure RPUF, M0, M0.1, M0.2 and M0.3 hydrogel-coated RPUF in open fire test.

| Hydrogel | Temperature (℃) | Time (day) | W_t_/W_0_ (%) |
| --- | --- | --- | --- |
| PAAm [1] | 25 | 10 | 30 |
| PAAAm-PDA [1] | 25 | 10 | 34 |
| PAAm-PDA-CaCl_2_ [1] | 25 | 10 | 80 |
| PVA/PA/MXene (This work) | 25 | 15 | 90 |

Table S1 The comparison of water maintenance of this work with other hydrogel coating.

| Number | Author | TTI (s) | HRR (kW/m^2^) | THR (MJ/m^2^) |
| --- | --- | --- | --- | --- |
| 1 | This work | 74 | 56.54 | 14.62 |
| 2 | Long Yuan et al. [2] | 25 | 101.50 | 2.20 |
| 3 | Ehsan N. Kalali et al. [3] | 23 | 282.00 | 83.10 |
| 4 | Selamawit Mamo Fufa et al. [4] | 22 | 70.00 | 68.00 |

Table S2 The comparison of flame-retardant wood of this work with other work.

# References

[1] Y. Huang, J. Zhou, P. Sun, L. Zhang, X. Qian, S. Jiang, C. Shi, Green, tough and highly efficient flame-retardant rigid polyurethane foam enabled by double network hydrogel coatings, Soft Matter, 17 (2021) 10555-10565.

[2] Long Yan, Zhisheng Xu, Nan Deng, Effects of polyethylene glycol borate on the flame retardancy and smoke suppression properties of transparent fire-retardant coatings applied on wood substrates, Progress in Organic Coatings, 135 (2019) 123-134.

[3] Ehsan N. Kalali, Lu Zhang, Marjan E. Shabestari, Jeremy Croyal, De-Yi Wang, Flame-retardant wood polymer composites (WPCs) as potential fire safe bio-based materials for building products: Preparation, flammability and mechanical properties, Fire Safety Journal, 107 (2019) 210-216.

[4] Selamawit Mamo Fufa, Anne Steen-Hansen, Bjørn Petter Jelle, Per Jostein Hovde, Reaction to fire and water vapour resistance performance of treated wood specimens containing TiO2 and clay nanoparticles, Fire and Materials, 38 (2014) 717-724.
